# Age-dependent NK cell dysfunctions in severe COVID-19 patients

**DOI:** 10.3389/fimmu.2022.1039120

**Published:** 2022-11-17

**Authors:** Cinzia Fionda, Silvia Ruggeri, Giuseppe Sciumè, Mattia Laffranchi, Isabella Quinti, Cinzia Milito, Paolo Palange, Ilaria Menichini, Silvano Sozzani, Luigi Frati, Angela Gismondi, Angela Santoni, Helena Stabile

**Affiliations:** ^1^ Department of Molecular Medicine, Sapienza University of Rome, Rome, Italy; ^2^ Istituto Pasteur-Fondazione Cenci Bolognetti, Rome, Italy; ^3^ Department of Public Health and Infectious Diseases, Sapienza University of Rome, Rome, Italy; ^4^ Istituto di Ricovero e Cura a Carattere Scientifico (IRCCS), Neuromed, Pozzilli, Italy

**Keywords:** Natural Killer cells, NK cell subsets, COVID-19, ageing, inflammation, T-BET, TGF-β

## Abstract

Natural Killer (NK) cells are key innate effectors of antiviral immune response, and their activity changes in ageing and severe acute respiratory syndrome coronavirus 2 (SARS-CoV-2) infection. Here, we investigated the age-related changes of NK cell phenotype and function during SARS-CoV-2 infection, by comparing adult and elderly patients both requiring mechanical ventilation. Adult patients had a reduced number of total NK cells, while elderly showed a peculiar skewing of NK cell subsets towards the CD56^low^CD16^high^ and CD56^neg^ phenotypes, expressing activation markers and check-point inhibitory receptors. Although NK cell degranulation ability is significantly compromised in both cohorts, IFN-γ production is impaired only in adult patients in a TGF-β−dependent manner. This inhibitory effect was associated with a shorter hospitalization time of adult patients suggesting a role for TGF-β in preventing an excessive NK cell activation and systemic inflammation. Our data highlight an age-dependent role of NK cells in shaping SARS-CoV-2 infection toward a pathophysiological evolution.

## Introduction

The current coronavirus disease 2019 (COVID-19) pandemic is caused by a new beta-coronavirus, namely severe acute respiratory syndrome coronavirus 2 (SARS-CoV-2) (1, 2). The clinical manifestations of COVID-19 are highly variable ranging from no or mild symptoms to severe disease requiring hospitalization and oxygen support (3, 4). Several studies investigated the impact of epidemiological factors and comorbidities on the asymptomatic, moderate, and severe forms of COVID-19 (5). In particular, the relevance of age-related processes in SARS-CoV-2 infection has been highlighted by a higher number of severe cases and death affecting older adults (6, 7, 8).

The excessive inflammation leading to a cytokine storm has been suggested to represent a key driver of COVID-19 immunopathogenesis and its evolution toward the more severe fatal form (9, 10).

The innate immune response represents the first line of host defense against viral infections, and the interaction between the immune system and host is crucial to restrict and eradicate the infection.

Since the discovery of Natural Killer (NK) cells as a population of innate lymphocytes endowed with a potent antitumoral and antiviral activity, our knowledge of innate lymphoid cells (ILCs) has rapidly expanded due to the identification of other ILCs, which are now classified into five distinct groups: NK cells, ILC1s, ILC2s, ILC3s and lymphoid tissue inducer (LTi). Except for NK cells, ILCs are rare in blood circulation but are enriched at mucosal surfaces where they play a crucial role in tissue homeostasis, inflammation and against pathogens (11, 12).

Natural Killer (NK) cells are a heterogeneous population of innate lymphoid cells (ILCs) that in humans were historically dissected into two main distinct phenotypic and functional subsets according to the expression levels of the adhesion molecule CD56 and the activating receptor CD16: the main cytotoxic CD56^dim^CD16^+^ population and the cytokine-producer CD56^bright^CD16^+/-^ subset (13). More recently, two additional subsets have been identified: the multifunctional CD56^low^CD16^low^ NK population and the dysfunctional CD56^neg^CD16^+^ NK cell subset, originally described in transplanted patients and in HIV-1 patients, respectively (13–15). Rare NK cell deficiency highlighted the important role of these innate lymphocytes in the control of several viral infections (16). In the early phase of viral infection, a number of cytokines, such as type I interferons, IL-12, IL-18, and IL-15, activate NK cells to kill virus infected cells and trigger their ability to produce anti-viral cytokines (17, 18),. As well, a complex array of activating and inhibitory receptors allows NK cells to recognize virus-infected cells and orchestrates their effector responses (19). Ageing does not change total NK cell number but significantly modulates NK cell subset frequency as well as their functional activity (20, 21).

NK cell compartment in elderly people is characterized by a shift toward the more mature CD56^dim^CD16^+^ and CD56^neg^CD16^+^ populations at the expense of the immature CD56^bright^ subset (22). Age affects NK cell cytotoxicity at single-cell level, and deeply impacts NK surface phenotype together with the ability to respond to cytokines, such as IL-2, IL-12, IFN-γ and IFN-α (23). Indeed, elderly NK cells display a reduced ability to produce IFN-γ in response to IL-2 stimulation (24, 25, 22, 26). Severe SARS-CoV-2 infected patients showed an activated NK cell phenotype, characterized by dysfunction or exhaustion, suggesting the importance of an adequate NK cell response to control disease progression (27–29). Moreover, an enrichment of NK cells in bronchoalveolar lavage samples from COVID-19 patients suggests their recruitment from blood to the site of infection (30, 31). Tissue NK cell migration is finely regulated by adhesion and chemotactic molecules and different NK cell subsets are endowed with distinct homing features due to a specific chemokine receptor profile. In this regard, during chronic inflammation or viral infections a preferential recruitment of CD56^bright^ NK cell subset to inflamed tissues is mediated by the CXCR3, CCR5 and CXCR6 chemokine receptors (32–34). Memory NK cells also emerged to play a role in the response against SARS-CoV-2 infection (27, 29, 35). Disease severity in COVID-19 patients was shown to positively correlate with an increased proportion of NK cell adaptive-like phenotype (NKG2C^+^FcRγ ^−/low^) enriched for type I interferon signaling (36), especially in aged individuals with COVID-19 (37).

Among the other ILC populations characterized over the last ten years, type 2 ILCs (ILC2) play crucial role in lung mucosal homeostasis as well as in asthma, chronic pulmonary diseases, and respiratory infections (38–40).

The transcription factor GATA-3 regulates ILC2 development and functions; several alarmins, including IL-33, IL-25 and thymic stromal lymphopoietin (TSLP), lead to ILC2 activation toward a type 2 response (41, 42). A significant decrease in circulating ILC2 frequency has been reported in severe COVID-19 patients, but not in moderate patients, alongside with an activated phenotype and an altered chemokine receptor profile (43). Consistently, a peripheral blood ILC2 expansion has been correlated with a better recovery from severe SARS-CoV-2 infection (44, 45).

Since age-dependent alteration of the immune system has been correlated with a higher risk of viral infections (46, 47, 48), in this study we longitudinally investigated the phenotypic and functional changes of NK cell compartment in severe SARS-CoV-2 infected adult and elderly patients requiring mechanical ventilation and the same pharmacologic treatment at hospital admission.

Our results depict a different phenotypic and functional scenario of the NK cell compartment in these two cohorts of patients, with the elderly patients showing a deep shift in NK cell subset distribution, despite an unaltered total NK cell number and frequency. In addition, we found more profound phenotypic changes in NK cells from the older patients, along with an unexpected high ability to produce IFN-γ in the early days after hospital admission. Since a longer and worse outcome of infection distinguished the elderly cohort of COVID-19 patients, our data suggest that an age-dependent impact on NK cell functionality has the potential to differently affect the outcome of SARS-CoV-2 infection.

## Materials and methods

### Ethics statement

The study was carried out in accordance with the principles of the Declaration of Helsinki and approved by the Ethics Committee of Sapienza University of Rome (approval number 5834, 0521/2020); informed assent/consent was obtained from healthy volunteers and patients.

### Patients

Twenty-seven SARS-CoV-2 infected patients admitted at Umberto I Hospital, Rome, Italy were recruited in the study from January to April 2021. The study inclusion criteria were: diagnosis of SARS-CoV-2 infection by PCR on nasopharyngeal swabs, disease severity, requirement of oxygen support at hospitalization and pharmacologic therapy. Patients were grouped into two distinct cohorts: adult (age < 65 years old) and elderly (age ≥ 65 years old). Age-matched healthy volunteers: age < 65 (n = 30) and age > 65 (n = 12) were enrolled in an anonymized form at Blood Transfusion Centre, Umberto I Hospital, Rome, Italy.

Peripheral blood collected in heparin tubes and plasma were sampled weekly starting from the hospital admission. The infection course was monitored until either discharge or death and clinical data were collected and used for statistical and correlation analyses. Patient’s clinical and demographic characteristics are reported in **Supplementary Table 1**.

### Multicolor immunofluorescence and flow cytometry

Freshly peripheral blood mononuclear cells (PBMC) were isolated from heparinized venous blood samples by lymphoprep (Ficoll-Hypaque, Cedarlane, Burlington, ON, Canada) density gradient centrifugation. For surface antigens, PBMCs were stained with specific monoclonal anti-human antibodies (mAbs) combined in appropriated panels and incubated for 20 min at 4 °C in the dark. A detailed list of mAbs used is reported in **Supplementary Table 2**. Fixable viability dye (BD Biosciences, Franklin Lakes, NJ, USA) was used to discriminate live and dead cells. For intracellular antigens (transcription factors, cytotoxic molecules and cytokines), PBMCs were fixed and permeabilized with the Foxp3/Transcription factor Staining kit (cat. #: 00-5523-00, eBioscience, Thermo Fisher, Waltham, MA, USA), according to manufacturer’s instructions, and then incubated for 40 min at 4 °C in the dark.

Sample acquisition was performed by LRSFortessa flow cytometer (BD Biosciences, San Jose, CA, USA) with 355-,405-, 488-, 561-, and 640-nm lasers.

### Functional assays

Freshly isolated PBMC from COVID-19 patients or healthy volunteers were used to perform functional assays.

CD107a degranulation assay: freshly isolated PBMC were co-cultured with MHC class I negative human erythroleukemia cell line K562 at 1:1 effector/target (E/T) ratio in a U-bottom 96-well tissue culture plate in complete medium at 37°C and 5% CO2 for 3 h in the presence of 50 μM Monensin (Golgi-stop; cat. #: M5273, Merck, Germany) for the last 2 h. PBMC alone were used as unstimulated controls. Following the incubation, cells were washed with 2% FCS, 0.05 mM EDTA PBS, and degranulation was assessed by evaluating CD107a expression in combination with surface and intracellular markers necessary to identify distinct NK cell subsets (49). In detail, co-culture was stained with Fixable Viability Stain, anti-CD3, anti-CD19, anti-CD14, anti-CD4, anti-CD5, anti-CD45, CD7, anti-CD56, anti-CD16, anti-EOMES, anti-TBET, anti-GATA3 and anti-CD107a.

IFN-γ production assay: freshly isolated PBMC were stimulated with IL-12 (20 ng/mL) plus IL-18 (25 ng/mL) (PeproTech, London, UK) at 37°C. After 1 h, 5 μg/mL Brefeldin A (cat. #: B7651, Merck, Germany) were added, and cells were incubated for an additional 5 h. At the end of stimulation, cells were washed with PBS and stained for surface antigens and subsequently fixed, permeabilized, and stained with anti-IFN-γ mAb in combination with intracellular markers (transcription factors). For the indicated experiments, PBMC were incubated with 20% of adult or elderly healthy donor plasma or with adult or elderly COVID-19 plasma, untreated or pre-treated for 30 min with anti-TGF-β blocking antibody (cat.#: MAB1835-SP R&D System, Minneapolis, USA). Upon 48h cells were stimulated with IL-12 (20 ng/mL) plus IL-18 (25 ng/mL) and assessed for IFN-γ production. In detail, PBMC were stained with Fixable Viability Stain, anti-CD3, anti-CD19, anti-CD14, anti-CD4, anti-CD5, anti-CD45, CD7, anti-CD56, anti-CD16, anti-EOMES, anti-TBET, anti-GATA3 and anti-IFN-γ.

### Plasma protein quantification

Blood plasma samples were isolated after whole blood centrifugation at 1,300 rpm for 10min at room temperature and stored at −80°C until analysis.

The chemokines and cytokines content of patient’s plasma was detected using Luminex technology custom human cytokine panel, including IL-21, IL-6, IL-13, IL-1β and TNF-α (R&D System, Minneapolis, USA) or ELISA kit assay for IL-10, IL-15, IP-10 (cat.#: 430601, cat.#: 435104, cat.#: 439904, ELISA MAX™ Deluxe Set Human Biolegend, San Diego, CA, USA) and for TGF-β (cat.#: DY1679-05, DuoSet ELISA R&D System, Minneapolis, USA) according to manufacturer’s instructions. Briefly, plasma samples were diluted 1:4 in dilution buffer provided with the kit. For Luminex technology, plasma samples were incubated for 30 min at room temperature with shaking with antibody-coated magnetic beads followed by an overnight incubation at 4 °C. All subsequent incubation steps were performed according to the manufacturer’s instructions. The assay plates were read using a Luminex MAGPIX system (Merck, Germany) and quantified using xPONENT analysis software (Luminex). To obtain the bioactive form of TGF-β, plasma was incubated with 1 N HCl followed by neutralization with 1.2 N NaOH according to the manufacturer’s instructions. The assay plates were read using VICTOR Multimode Microplate Reader (PerkinElmer Akron, Ohio, USA).

### K-means clustering on clinical and experimental data

To stratify our cohort of patients, we utilized the percentage of IFN-γ of the NK cell subsets and their hospitalization time on anonymized patients to perform a K-means clustering in R (version 4.0.2). In order to unbiasedly classify our dataset we excluded the age parameter from our calculation. Briefly, the patient’s data (**Supplementary Table S3**) were firstly re-organized by PCA with the R package “rpca” (version 0.2.3). With the package FactoMineR (version 2.4) we then scaled the data and identified the optimal cluster number (n=2) with the “silhouette” method and compute the K-means with 100 permutations.

### Statistical analysis

For statistical analysis GraphPad Prism v8 was used (GraphPad, San Diego, CA, USA). The following nonparametric tests were used: Two-way analysis of variance (ANOVA) (Tukey’s multiple comparisons test), One way ANOVA (Kruskal-Wallies test multiple comparisons) and Mann Withney test.

## Results

### Study design and clinical cohorts

**Figure 1:**
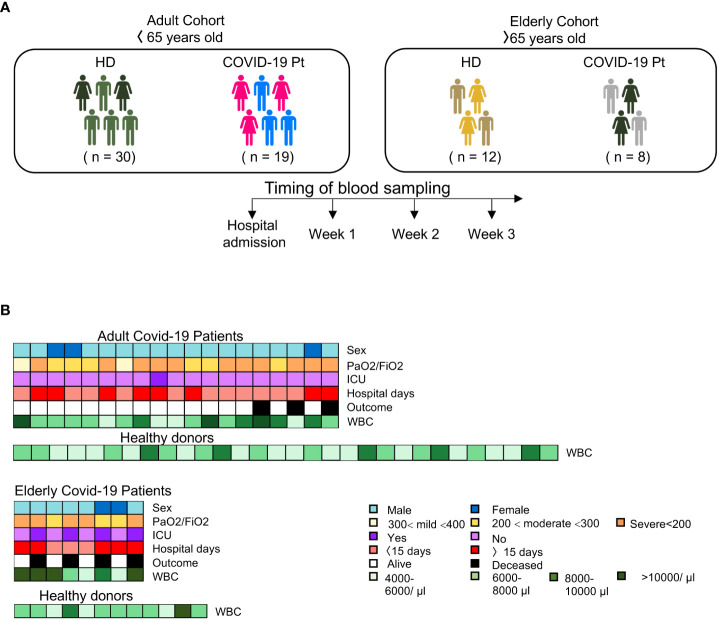
Overview of study design. **(A)** Experimental design and workflow of the study. **(B)** Schematic representation of the major demographic (age and sex) and clinical data [PaO2/FiO2, intensive care unit (ICU), hospital days, white blood count (WBC) and outcome] of patients enrolled in the study.

To profile the immunophenotype of NK cells in patients affected by SARS-CoV-2 infection, we longitudinally evaluated 27 severe COVID-19 patients who have been admitted to Umberto I Hospital in Rome. Patients were stratified into two groups, below and over 65 years of age. As a control, forty-two age-matched healthy volunteers were also recruited (**Figure 1A**). The median age of the elderly group was 83 ± 9,08 years while the adult group was 49.5 ± 10.6 years of age; the patients of both cohorts were mainly male (81%), 5 elderly and 3 adult patients showed significant comorbidities (hypertension, diabetes or obesity), and all required oxygen therapy, mechanical ventilation and the same pharmacologic treatment at hospital admission. A detailed description of patient clinical characteristics is presented in **Supplementary Table 1** and a graphical overview summarizing the major demographic and clinical data of patients is shown in **Figure 1B**. The average of hospitalized days for adult patients was 15.5 ± 9.1 while for elderly was 33.5 ± 3.5; moreover the 50% of the over-65 patients required intensity care unit (ICU) treatment and 50% of them died. Differently, no under-65 patients died or experienced ICU and 63% of them were discharged within 15 days.

### SARS-CoV-2 infection differently impacts NK cells in adult and elderly patients

**Figure 2:**
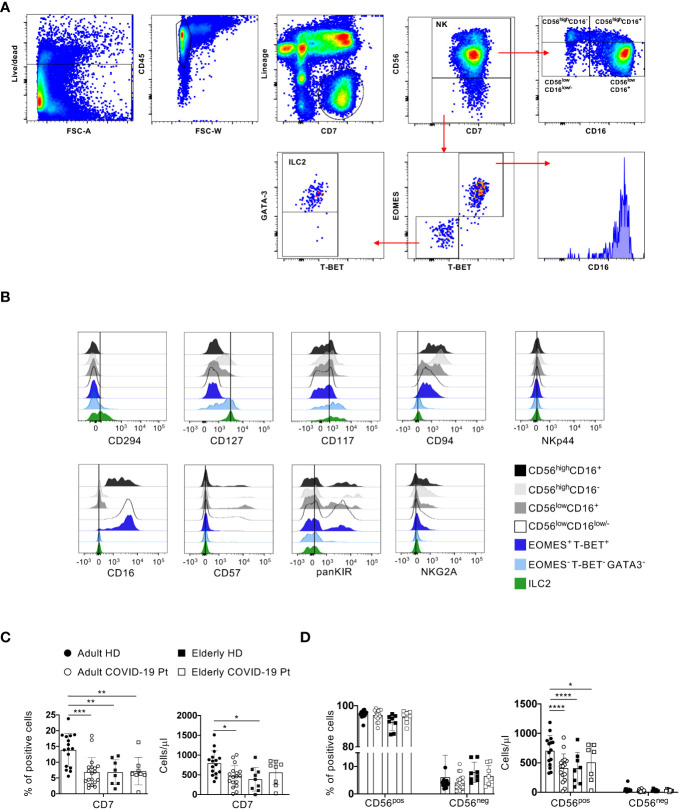
Changes in adult and elderly innate lymphocytes and NK cells during SARS-CoV-2 infection. **(A)** Gating strategy used to identify conventional NK cells (NK), CD56^neg^EOMES^+^/T-BET^+^ cells and ILC2. NK and EOMES^-^/T-BET^-^ cells were gated on CD56^pos^ and CD56^neg^ NK cell populations respectively within Lin^-^ (CD3, CD4, CD5, CD14, CD19) CD45^+^CD7^+^ cells. ILC2 were gated on EOMES^-^/T-BET^-^ as GATA3^+^. Representative overlays displaying CD127, CD117 and CD294 (as ILC2 markers). **(B)** Representative overlays displaying CD127, CD117 and CD294 (as ILC2 markers) as well as CD16, CD57, KIRs and NKG2A (as NK cell markers) expression on the indicated cell populations in a healthy donor. **(C, D)** Histograms represent the median ± SD of the frequency and the total cell count/μl of the indicated cell populations in adult (n = 19) and elderly (n = 8) patients (Pt) compared to age-matched volunteers (HD; adult = 16, elderly = 9). * p< 0.05, ** p< 0.01, *** p< 0.001, **** p< 0.0001. One way ANOVA Kruskal-Wallies test multiple comparisons).

To provide an in-depth characterization of NK cell compartment in the early days after hospital admission, we employed an 18-color flow cytometry panel on freshly isolated peripheral blood mononuclear cells (PBMC) from severe COVID-19 patients and age-matched healthy controls. Firstly, to investigate NK cell compartment, we used a manual gating strategy of flow cytometry data to identify CD56^pos^ and CD56^neg^ NK cells among lineage negative (CD3, CD4, CD5, CD14, CD19) CD45^+^CD7^+^ innate lymphocytes (50). According to the expression levels of CD56 and CD16 we further dissected the CD56^pos^ cells into four subsets: CD56^high^CD16^+^, CD56^high^CD16^-^, CD56^low^CD16^+^ and CD56^low^CD16^low/-^, while the un-conventional NK cell population was identified within the CD56^neg^ cells as EOMES, T-BET and CD16 positive cells (**Figure 2A**).

Finally, ILC2 were identified within EOMES^-^T-BET^-^ CD56^-^ cells as GATA-3^+^ also expressing additional markers defining this subpopulation of ILC, such as CD127, CD117 and CD294 but not NK cell receptors, such as CD16, CD57, KIRs and NKG2A (**Figures 2A, B**).

Compared to adult controls, a significant decrease of the frequency and the absolute number of CD7^+^ population as well as of total cell count of CD56^pos^ cells was observed in infected adults and in both elderly healthy controls and patients. However, these differences did not emerge between older individuals with COVID-19 and age-matched healthy controls (**Figures 2C, D**).

These findings indicate a basal difference in the content of the absolute number of innate lymphocytes and NK cells between adult and elderly healthy individuals as well as a diverse impact of SARS-CoV-2 infection on these populations in adult and elderly patients as compared to age-matched controls.

### Alterations of NK cell subsets in adult and elderly COVID-19 patients

We next analyzed CD56^pos/neg^ cells by using manual gating strategy. In detail, on the basis of CD56 surface density, we discriminated CD56^pos^ cells in CD56^high^ and CD56^low^. Because of their CD16 expression, both these populations were further dissected in two subsets, CD56^high^CD16^+^ and CD56^high^CD16^-^, and CD56^low^CD16^+^ and CD56^low^CD16^low/-^.

**Figure 3:**
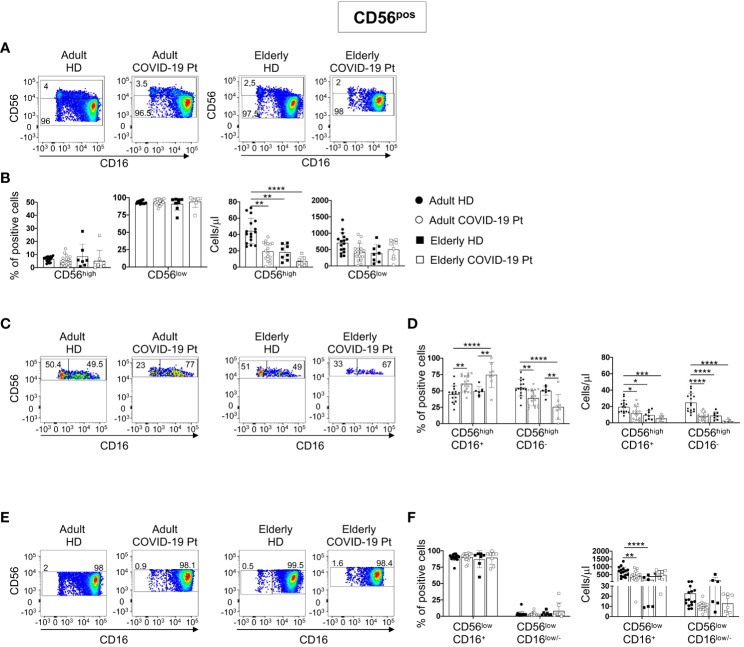
Alterations in circulating CD56^pos^ cells in adult and elderly COVID-19 patients at hospital admission. Gating strategies used to identify total and relative frequency of CD56^high^ and CD56^low^ cell populations and their subsets are shown on a representative adult c COVID-19 patient and age-matched controls **(A, C, E)**. CD56^high^ and CD56^low^ cell populations were gated on Lin^-^ CD45^+^CD7^+^ CD56^+^ and distinguished based on CD16 expression. Frequency and total cell count/μl of total CD56^high^ and CD56^low^ cell **(B)** as well as of specific subsets CD56^high^CD16^+^ and CD56^high^CD16^-^ **(D)**, and CD56^low^CD16^+^ and CD56^low^CD16^low^/^-^ **(F)** in COVID-19 patients and healthy donors are shown. Histograms show mean ± SD. * p< 0.05, ** p< 0.01, *** p< 0.001, **** p< 0.0001. Two-way analysis of variance (ANOVA) Tukey’s multiple comparisons test and One way ANOVA Kruskal-Wallies test multiple comparisons.

Aged individuals had a count of CD56^high^ cells lower than adults, but it remained unaltered in infected patients. Differently, SARS-CoV-2 infection significantly reduced the absolute number of CD56^high^ in adults (**Figures 3A, B**).

Within the CD56^high^, loss of CD56^high^CD16^-^ NK cells was accompanied by an increase of CD56^high^CD16^+^ subset, and this feature was common to elderly and adult patients as compared to age-matched controls (**Figures 3C, D**).

Although no relevant changes characterized the frequency and the absolute number of CD56^low^ population, in both cohorts we found a different trend for the total count of CD56^low^CD16^+^ cells, with a reduction in adult patients and an increase in elderly patients as compared to age-matched controls. Moreover, in all patients, the absolute number of the multifunctional CD56^low^CD16^low/-^ NK cell subset showed a tendency to decrease (**Figures 3E, F**).

These observations indicate that NK cell subsets resulted differently altered in elderly and adult SARS-CoV-2 infected patients.

**Figure 4:**
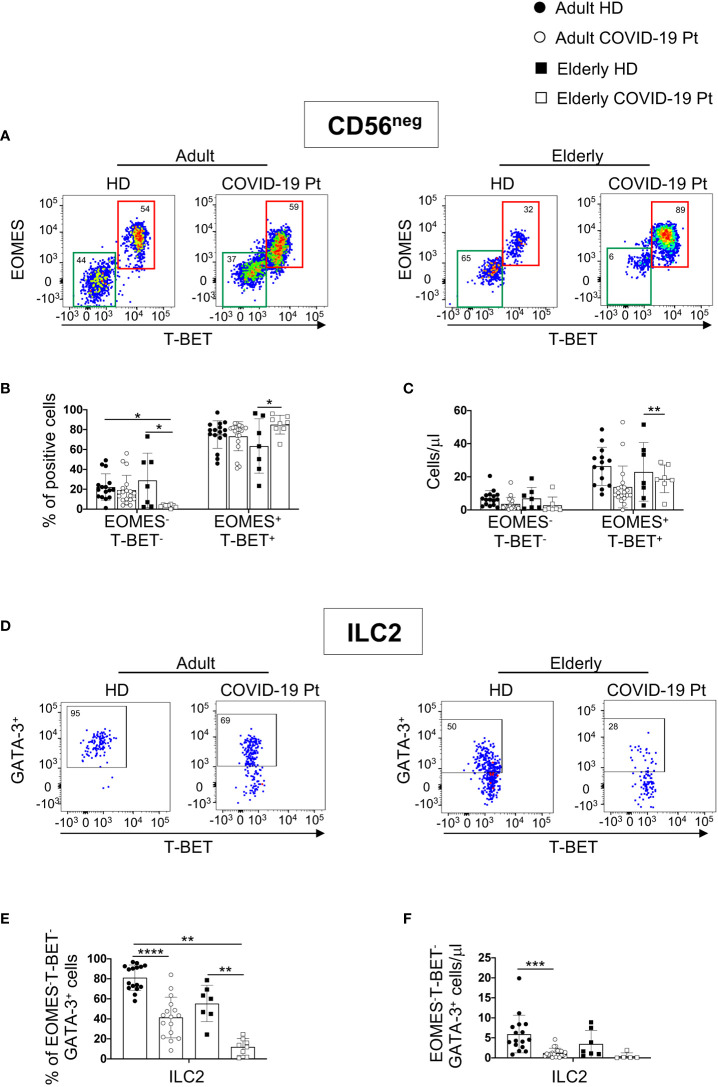
Alterations in circulating CD56^neg^ cells in adult and elderly COVID-19 patients at hospital admission. Gating strategies used to identify EOMES^-^ T-BET^-^ and EOMES^+^ T-BET^+^ subsets within CD56^neg^ cells **(A)** and ILC2 among EOMES^-^ T-BET^-^ cells **(D)** and their relative frequency are shown on a representative adult and elderly COVID-19 patient and age-matched controls. Lin^-^ CD45^+^CD7^+^CD56^-^ cells were analyzed for the expression of transcription factors EOMES and T-BET. ILC2 were identified within EOMES^-^/T-BET^-^ CD56^-^ cells as EOMES^-^T-BET^-^ GATA3^+^. Percentage and absolute counts of CD56^-^ EOMES^-^/T-BET^-^ and EOMES^+^ T-BET^+^ **(B, C)** and ILC2 **(E, F)** are reported. Histograms show mean ± SD. * p< 0.05, ** p< 0.01, *** p< 0.001, **** p< 0.0001. Two-way analysis of variance (ANOVA) Tukey’s multiple comparisons test and One way ANOVA Kruskal-Wallies test multiple comparisons.

Following separation of CD56^neg^ population in two distinct subsets, based on EOMES and T-BET expression, we revealed additional differences in the two groups of COVID-19 patients (**Figure 4A**). In particular, in elderly patients a significant decrease in the frequency of EOMES^-^ T-BET^-^ cells paralleled an enrichment in the proportion of the un-conventional EOMES^+^ T-BET^+^CD16^+^ cell subset. By contrast, in adult patients only the total cell count of un-conventional NK cells was reduced (**Figures 4B, C**).

In agreement with previous studies reporting a negative correlation of circulating ILC2 frequency and disease severity (27, 51), in the context of EOMES^-^ T-BET^-^ cell population, we observed a significant contraction of the EOMES^-^ T-BET^-^ GATA-3^+^ cell subset frequency in both cohorts of patients (**Figures 4E, F**).

These findings demonstrate that, within CD56^neg^ population, substantial variations in the frequency of two subsets EOMES^-^ T-BET^-^ and EOMES^+^ T-BET^+^CD16^+^ occurred mainly in elderly patients, but ILC2 decreased in both cohorts of infected individuals.

A longitudinal analysis also highlighted differences between the two groups of patients.

**Figure 5:**
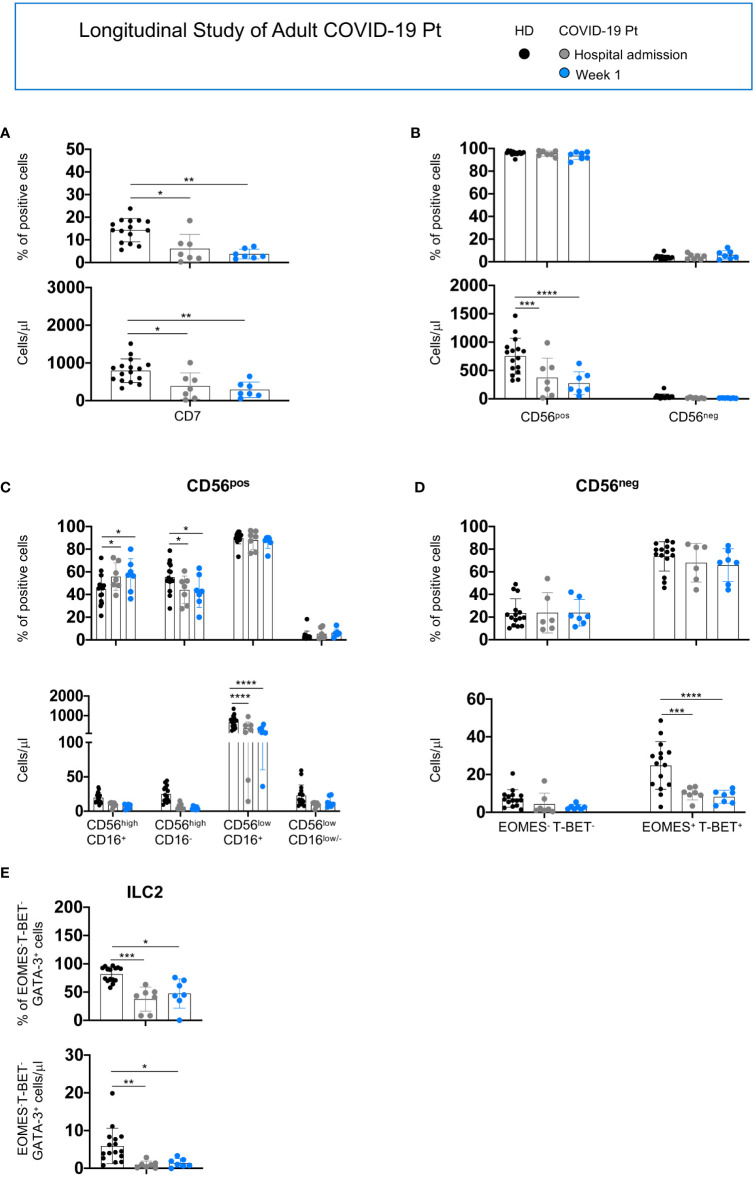
Longitudinal analysis of NK/ILC populations in adult COVID-19 patients. Histograms represent the mean ± SD of the frequency and the absolute count of CD7^+^ **(A)**, CD56^pos^ and CD56^neg^ **(B)**, CD56^high^ **(C)** and CD56^low^ **(D)** and ILC2 **(E)** in adult patients (n = 7) at 1week post-hospitalization. Cell populations were gated as described above. * p< 0.05, ** p< 0.01, *** p< 0.001, **** p< 0.0001. Two-way analysis of variance (ANOVA) Tukey’s multiple comparisons test and One way ANOVA Kruskal-Wallies test multiple comparisons.

For adult patients hospitalized longer than 1 week, we found a persistent reduction of CD7^+^, CD56^pos^, CD56^low^CD16^+^, CD56^neg^ and ILC2 populations from the hospital admission to discharge (**Figures 5A–E**).

**Figure 6:**
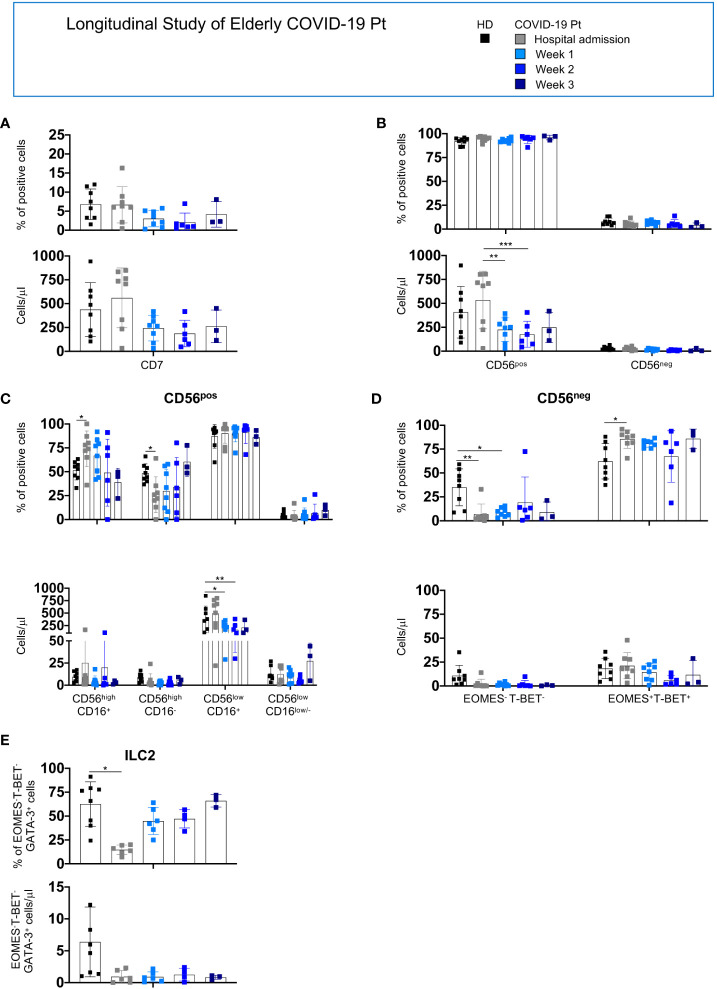
Longitudinal analysis of NK/ILC populations in elderly COVID-19 patients. Histograms represent the mean ± SD of the frequency and the absolute count of CD7^+^ **(A)**, CD56^pos^ and CD56^neg^ **(B)**, CD56^high^ **(C)** CD56^low^ **(D)** and ILC2 **(E)** in elderly patients at the indicated period. Cell populations were gated as described above. Elderly patients: hospital admission n = 8; week 1 n = 8; week 2 n = 6; week 3 n = 3. * p< 0.05, ** p< 0.01, *** p< 0.001. Two-way analysis of variance (ANOVA) Tukey’s multiple comparisons test and One way ANOVA Kruskal-Wallies test multiple comparisons.

For the elderly patients, we observed that the absolute number of CD56^pos^ cells decreased at 1week post-hospitalization while the CD56^high^CD16^+^, CD56^high^CD16^-^ subsets and ILC2 were altered at hospital admission; however, all populations tended to recover with a frequency comparable to age-matched controls at discharge (**Figures 6A–E**).

Collectively, SARS-CoV-2 infection differently affects NK cell compartment in adult and elderly patients, leading to severe reduction of total NK cells as well as of all NK cell subsets only in adult patients. Moreover, a reduction of ILC2 characterizes both cohorts. These alterations persisted in adult patients still hospitalized at day 7. Indeed, no major changes were observed in CD56^pos^ and CD56^neg^ total count and frequency in the elderly cohort; however, a peculiar skewing among the CD56^pos^ and CD56^neg^ populations characterized these patients at hospital admission and progressively disappeared during infection recovery. These observations highlighted that changes in the distribution or absolute number of NK cell subsets do not correlate with the outcome of infection.

### NK cell degranulation ability is impaired in adult and elderly severe COVID-19 patients

**Figure 7:**
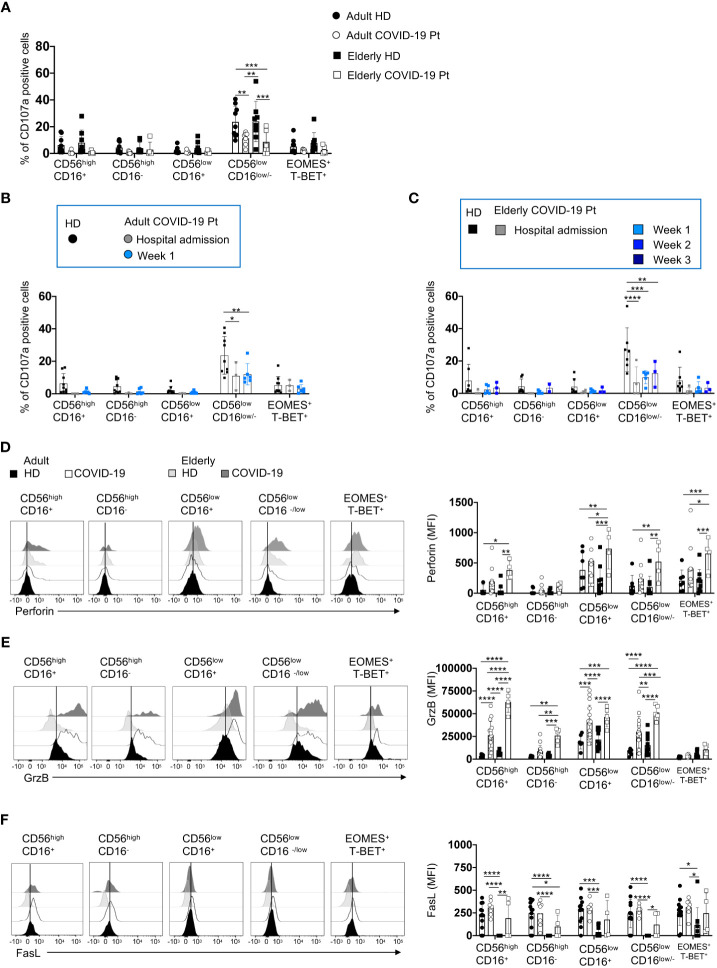
An impairment of degranulation ability characterizes both adult and elderly NK cell subsets. The percentage of K562 target cell-induced CD107a positive NK cell subsets from adult and elderly COVID-19 patients and healthy controls is shown early after hospital admission **(A)** and at different times after hospitalization in adult **(B)** and elderly **(C)** patients. Representative overlays and mean fluorescence intensity (MFI) of Perforin **(D)**, Granzyme B (GrzB) **(E)** and FasL **(F)** expression by distinct NK cell subsets are reported for COVID-19 patients and age-matched controls. Histograms represent the mean ± SD. * p< 0.05, ** p< 0.01, *** p< 0.001, **** p< 0.0001. Two-way analysis of variance (ANOVA) Tukey’s multiple comparisons test and One way ANOVA Kruskal-Wallies test multiple comparisons.

Since the adult group was characterized by a faster viral clearance accompanied by a better outcome of the disease, we investigated the effector functions of the different NK cell subsets within the CD56^pos^ and CD56^neg^ populations. To this aim, we performed a degranulation assay and evaluated the proportion of CD107a positive CD56^high^CD16^+/−^and CD56^low^CD16^+/−^ NK cells once co-cultured with HLA class-I deficient K562 target cells. We observed that the multifunctional CD56^low^CD16^low/-^ NK cell subset was significantly compromised in the ability to degranulate upon target cell contact in both cohorts of patients, at the early days of infection (**Figure 7A**), and such functional defects persisted until hospital discharge (**Figures 7B, C**).

However, we did not find a deficit in the cytotoxic machinery. In line with recent reports (27, 52, 53), we observed an increase of granzyme B and perforin, but not of FasL expression, that was more pronounced in the elderly patients (**Figures 7D–F**).

**Figure 8:**
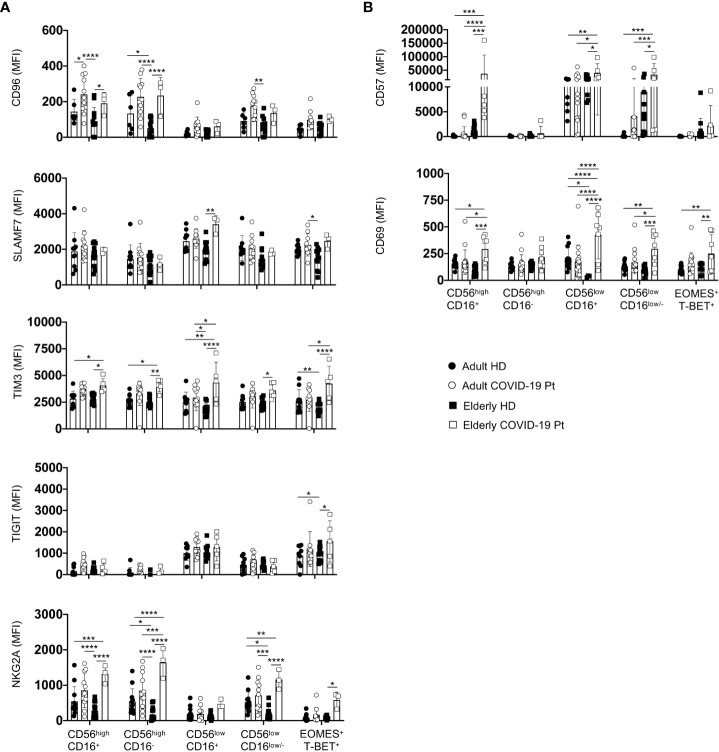
Receptor prolife of adult and elderly NK cell subsets. Expression levels of inhibitory **(A)** and activation/maturation **(B)** markers on NK cell subsets in adult and elderly COVID-19 patients alongside healthy controls early after hospital admission. MFI median values ± SD for each marker are shown. * p< 0.05, ** p< 0.01, *** p< 0.001, **** p< 0.0001. Two-way analysis of variance (ANOVA) Tukey’s multiple comparisons test and One way ANOVA Kruskal-Wallies test multiple comparisons.

Since NK cell functional impairment is often associated with increased expression of check-point inhibitory receptors along with an altered state of activation (27), we performed a phenotypic analysis using a selective panel of inhibitory receptors and activation/maturation markers (54). We found phenotypic alterations within NK cells of elderly patients (**Figure 8**). Consistently, we detected a significant increase in the expression levels of the inhibitory receptors SLAMF7, TIM3, CD96, NKG2A and TIGIT on the NK cell subsets from elderly patients compared with adult and elderly healthy controls, indicating that NK cell functionality could be deeply compromised (**Figure 8A**). In addition, an activated and more mature phenotype also strictly characterized NK cells from elderly patients as highlighted by the higher expression of the CD69 and CD57 markers as compared to aged-matched controls as well as adult patients (**Figure 8B**). This phenotype was associated with a plasma content of the inflammatory cytokines IP-10, IL-10, IL-13, IL-6 and TNF-α higher in SARS-CoV-2 infected elderly than in adult patients (**Supplementary Figure S1**), supporting that the long hospitalization time for these patients might be due to more complex alterations of NK cell functionality.

### NK cell ability to produce higher levels of IFN-γ discriminates elderly from adult severe COVID-19 patients

**Figure 9:**
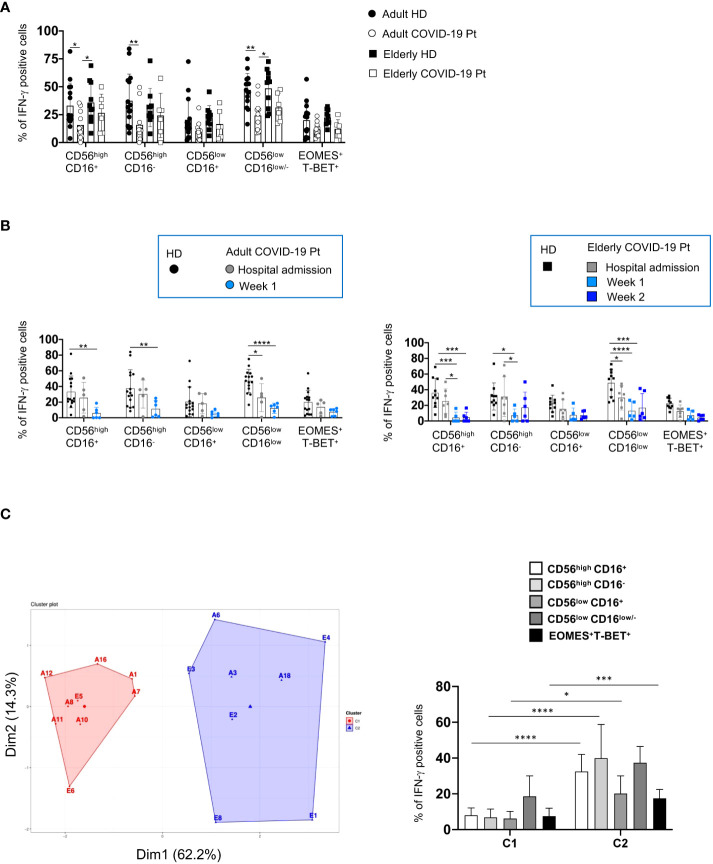
Differential ability of producing IFN-γ by adult and elderly COVID-19 NK cell subsets. The frequency of IFN-γ positive cells, upon IL-12 plus IL-18 stimulation, is shown for each NK cell subset from adult and elderly COVID-19 patients compared with healthy controls early after hospital admission **(A)** and at different times after hospitalization in adult and elderly patients **(B)**. Histograms represent the mean ± SD. K-means clustering of our patient cohort identified two clusters based on IFN-γ production by NK cell subsets and the hospitalization time (**Supplementary Table S3**) (left panel) **(C)**. These two clusters differentially present the indicated population for IFN-γ production (right panel). *p< 0.05, **p< 0.01, *** p< 0.001, **** p< 0.0001 . Two-way analysis of variance (ANOVA) and Sidak’s multipletest.

To gain functional insights on CD56^pos^ and CD56^neg^ cells in the two cohorts of COVID-19 patients, we also assessed the ability of distinct NK cell subsets to produce IFN-γ. At hospital admission, a significant impairment in IL-12 plus IL-18 induced-IFN-γ production characterized adult NK cells, while elderly NK cells retained the ability to produce this cytokine (**Figure 9A**). Based on these observations, we wondered whether the IFN-γ production was impaired also in adult COVID-19 patients who have undergone a longer period of hospitalization. Intriguingly, in these patients NK cells did not show an early defective IFN-γ production but, similarly to elderly patients, they displayed a cytokine functional impairment starting from the second week of hospitalization (**Figure 9B**).

Accordingly, an unbiased analysis of correlation between IFN-γ production by NK cell subsets and hospitalization time of COVID-19 patients also resulted in their separation in two different clusters corresponding to adult and elderly group (**Figure 9C** and **Supplementary Table 3**).

**Figure 10:**
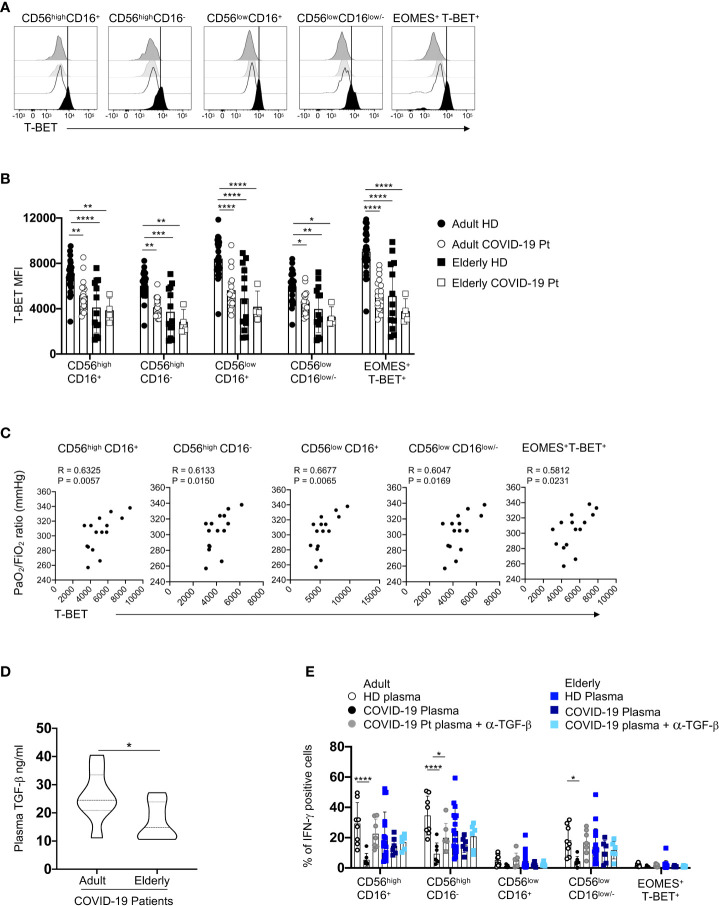
TGF-β regulates IFN-γ production in adult COVID-19 NK cell subsets. Representative overlays displaying T-BET expression of distinct NK cell subsets from adult and elderly COVID-19 patients and age-matched controls **(A)**. Summary of data showing the median values ± SD of T-BET MFI expression on each subset **(B)**. Spearman’s rank correlation of T-BET expression (MFI) and the oxygenation index PaO2/FiO2 ratio at hospital admission in adult patients **(C)**. Plasma levels of TGF-β of elderly and adult COVID-19 patients at hospital admission (adult = 12, elderly = 7). * p< 0.05 unpaired t-student test analysis is indicated **(D)**. PBMC from healthy donors were cultured in medium containing adult healthy or COVID-19 plasma or elderly healthy or COVID-19 plasma for 48h in the absence or in the presence of α-TGF-β blocking antibody. The percentage of IFN-γ positive cells after IL-12 plus IL-18 stimulation was assessed for each NK cell subset. Histograms represent the mean ± SD **(E)**. * p< 0.05, ** p< 0.01, *** p< 0.001, **** p< 0.0001. Two-way analysis of variance (ANOVA) Tukey’s multiple comparisons test and One way ANOVA Kruskal-Wallies test multiple comparisons.

Interestingly, a significant reduction of T-BET expression, a well-known regulator of IFN-γ production in NK cells, marked only adult NK cell patients (**Figures 10A, B**); moreover, the mean fluorescent intensity of T-BET positively correlated with the oxygenation index PaO_2_/FiO_2_ ratio (arterial oxygen partial pressure/fractional inspired oxygen), which is a clinical parameter used to evaluate the pulmonary functionality (**Figure 10C**, 55). For elderly group, healthy individuals displayed lower levels of T-BET than adult controls, but the infection did not affect the expression of this transcription factor.

To identify possible mechanisms underlining this impaired IFN-γ production, we focused on TGF-β, a cytokine produced during the early phase of SARS-CoV-2 infection and able to modulate T-BET expression (52, 56). We found that TGF-β plasma content of adult COVID-19 patients was higher than elderly patients, suggesting that TGF-β expression accounts for the defective IFN-γ production by adult NK cells in a T-BET-dependent way (**Figure 10D**). Accordingly, plasma from adult COVID-19 patients, but not that from elderly patients, significantly reduced IFN-γ production by healthy donor NK cells and anti-TGF-β blocking antibody reverted this inhibitory effect, thus demonstrating a crucial role of this cytokine in these regulatory mechanisms (**Figure 10E**).

These findings suggest that, in elderly COVID-19 patients, a high production of IFN-γ due to an early excessive NK cell activation could amplify the systemic inflammatory response with a poor prognosis. Differently, in adult patients, TGF-β may contribute to limit IFN-γ secretion by NK cells leading to a favorable outcome.

## Discussion

Several studies have investigated NK cell compartment in SARS-CoV-2 infected patients with a different spectrum of clinical manifestations (53), however our understanding of the potential impact of age-related differences on NK cell compartment in severe adult and elderly COVID-19 patients remains limited (37, 57)

In line with other viral infections, such as hantavirus or dengue fever disease, a reduction of NK cell number has been reported in SARS-CoV-2 infection (17, 18, 58). An alteration of NK cell compartment consisting in a shift toward a more mature phenotype of NK cells, due to an enrichment of the CD56^low^CD16^high^ subset accompanied by the reduction of CD56^high^CD16^-/+^ one, especially in severely ill COVID-19 patients, has also been described by several studies (27). Both an exhausted or an adaptative phenotype was attributed to the expanded CD56^low^CD16^high^ NK cells depending on severity of the infection and cohort clinical and demographic characteristics (28, 35, 59). Moreover, these changes of the CD56^high^ NK cell subset have been associated to multiple and non-mutually exclusive causes, including its preferentially recruitment from the blood into the inflamed lungs, an accelerated maturation toward the CD56^low^CD16^high^ subset or a lower resistance to cytokine-induced apoptosis (29, 60).

Our study highlights a different picture of NK cell population in the adult and elderly cohort of patients all requiring mechanical ventilation at hospital admission but characterized by different outcome. A significant decrease of total innate CD7^+^ lymphocytes characterized only the adult COVID-19 patients with a general impact on all CD56^pos^ cells and on the frequency of the main NK cell subsets CD56^high^ and CD56^low^CD16^high^. Differently, elderly individuals with COVID-19 showed only little changes in the total count and frequency of innate CD7^+^ lymphocytes or NK cells. Moreover, aged individuals had a count of CD56^high^ cells lower than adults and COVID-19 infection induced a decline in the percentage of this subset alongside with an increase of CD56^low^CD16^high^ population with respect to age-matched volunteers.

Interestingly, a common feature to both groups of COVID-19 patients was represented by a switch in the frequency of the CD16^+^ and CD16^-^ subsets within CD56^high^ NK cells, thus suggesting a promotion of CD56^high^CD16^-^ cell maturation toward CD56^high^CD16^+^ NK cell subset. Indeed, the CD56^high^CD16^+^ cells have been described as a phenotypic and functional intermediate between CD56^high^CD16^-^ and CD56^dim^CD16^+^ cells, endowed with cytolytic activity and their accumulation has been also associated with aging (61, 62),Moreover, loss of CD56^high^CD16^-^ may also reflect their migration to the inflamed lungs accordingly to their high expression of the chemokine receptor CXCR3, CXCR6 and CCR5 and also supported by the increased concentration of the ligands for these chemokine receptors observed in bronchoalveolar lavage of COVID-19 patients (30, 32, 33, 63).

Noteworthy, a deep loss of circulating ILC2 population characterized both cohorts at hospital admission in line with previous studies correlating the low circulating ILC2 levels with a more severe COVID-19 disease (43, 44). ILC2 are effectors of type two responses by recruiting eosinophils during allergic inflammation and viral lung infections upon the release of alarmins by damaged epithelial cells (38, 39). This innate lymphoid population has been proven to be crucial in the resolution of inflammation and tissue repair by amphiregulin secretion (41). Gomez-Caneda et al. reported a protective role for NKG2D^+^ ILC2 subset in severe SARS-CoV-2 infection, showing that a higher frequency of these circulating innate cells positively correlated with reduced hospitalization and requirement of mechanical ventilation (64). Of note, the recovery of SARS-CoV2 infection was timely associated with an increase of CCR10^+^ILC2 and resolution of oxygenation impairment, supporting a strong correlation between the frequency of PB ILC2 and recovery of lung functions (44). Consistently, despite the respiratory insufficiency at the hospital admission, we detected a significant higher frequency of ILC2 in the PB of adult COVID-19 cohort compared to elderly patients, supporting a positive effect of these innate lymphoid cells in the faster resolution of viral infection and promotion of lung recovery in this cohort. Notably, a progressively recovery of ILC2 frequency also characterized the elderly patients when monitoring at the last time before the discharge.

The surface expression of CD56 and CD16 molecules has been historically used to identify functionally distinct NK cell subsets, including the first characterized immunomodulatory CD56^high^CD16^-/+^ and the major cytotoxic CD56^dim^CD16^+^ NK cell populations together with the more recently discovered multifunctional CD56^low^CD16^low^ and dysfunctional CD56^neg^CD16^high^ NK cell subsets (14, 15, 65, 66), The un-conventional CD56^neg^ subset was firstly identified in chronic HIV-1 infection and its expansion has been shown to be common to other chronic viral infections including hepatitis C virus (HCV), cytomegalovirus (CMV) and Epstein Bar virus (EBV) infection (67–69). In healthy individuals this population shows a phenotype similar to CD56^dim^CD16^+^ NK cell subset together with a common EOMES^pos^ T-BET^high^ transcription factor profile, but it is less cytotoxic while retains a comparable IFN-γ production ability upon cytokine stimulation (15). However, the expanded CD56^neg^ subset in viral infected individuals exhibits an impaired cytotoxicity and a reduced ability to produce IFN-γ and TNFα, resembling an exhausted phenotype. Intriguingly, a more prominent expansion of this dysfunctional population has been described in elderly CMV and EBV co-infected individuals and it has been correlated with an immune risk profile (70). In our study, we also explored whether SARS-CoV-2 infection could affect the CD56^neg^ compartment and the expression levels of the transcription factors EOMES and T-BET allowed us to better dissect this cell population. Notably, we observed an increase of EOMES^+^T-BET^+^CD56^neg^ NK cell frequency only in the elderly patients. Because of differences in the NK population between adult and elderly severe patients, we can speculate that ageing has a great impact in skewing NK cell subsets leading to the worst outcome in the elderly COVID-19 patients.

The antiviral role of NK cells can be mediated by direct cytotoxicity or through IFN-γ production able to directly interfere with viral infection or to promote monocyte and macrophage activation as well as type I mediated responses. The degranulation ability against K562 target cells of the multifunctional CD56^low^CD16^low^ NK cell subset was strongly compromised in both cohorts of COVID-19 patients at hospital admission and persisted until discharge. In keeping with previous observations, the functional NK cell impairment did not correlate with a reduction in the cytolytic machinery, but rather almost all NK cell subsets have a higher cell content of granzyme B or perforin (27). Although the degranulation impairment characterized both cohorts of patients, only NK cells of elderly patients showed a suppressor phenotype with an elevated expression of several inhibitory receptors. Accordingly, mounting evidence demonstrated that high levels of immune check-point receptors strongly compromise NK cell functionality (27, 29, 71). Furthermore, elderly NK cells displayed a more activated and mature phenotype. Taken together, these observations indicate a profound functional alteration of NK cell compartment in elderly patients that could explain their longer and more severe outcome of SARS-CoV-2 infection. Along this line, we were surprised to observe that NK cells from elderly COVID-19 patients, unlike those of adults, were still capable to produce IFN-γ following cytokine stimulation in the early days after hospital admission, while their functionality declined progressively in the later weeks. We can hypothesize that early NK cell hyperactivation contributes to promote myeloid cell activation and the consequent cytokine-storm that causes the more severe COVID-19 symptomatology of in elderly patients. Consistently, a higher level of some inflammatory cytokines, such as IL-6, IL-13, TNF-α, IL-10 and IP-10, was detected in the plasma of elderly with respect to adult patients at hospital admission. In this regard, it is interesting to note that adult patients with a slower recovery from SARS-CoV-2 infection showed the same IFN-γ dysfunctional kinetics as elderly patients. A dysregulated inflammatory state caused by an hyperactivated innate immune-response emerged as a key factor determining the divergent evolution of SARS-CoV-2 infection, ranging from asymptomatic to severe and often lethal outcome (10, 35). Intriguingly, we observed a lower expression level of the transcription factor T-BET in adult patients with respect to age-matched volunteers while no remarkable changes were observed in the elderly cohort with respect to their controls. Since T-BET is a key regulator of IFN-γ production by NK cells, we suggest that this transcription factor may be responsible for the reduced ability of adult NK cells to produce this cytokine (72, 73). A recent study reported a crucial role of untimely TGF-β production in severe COVID-19 patients leading to NK cell cytotoxic impairment by reducing T-BET expression (52). Consistently, we detected higher levels of TGF-β in adult patient plasma compared to elderly in the early days of SARS-CoV2 infection suggesting that it contributes to reduced IFN-γ production by adult NK cells *via* T-BET modulation. Differently, we did not observe any significant difference in the production of IFN-γ, T-BET and TGF-β levels between survived and death elderly patients (data not shown) suggesting a role for this pathway in the control of inflammatory response in the early phase of infection. Of note, in mouse T-BET^-/-^ NK cells early IFN-γ production was not affected, however T-BET expression was required for the maintenance of IFN-γ secretion by NK cells (55). Thus, we can speculate that TGF-β/T-BET axis plays a key role in limiting an excessive NK cell activation that could amplify the systemic inflammatory response; consistently, higher TGF-β may prevent an excessive dysfunction of NK cells in adult patients leading to a less severe infection course.

In conclusion, our findings reveal that an age-dependent impact on NK cell functionality may differently affect the outcome of SARS-CoV2 infection.

## Data availability statement

The original contributions presented in the study are included in the article/**Supplementary Materials**. Further inquiries can be directed to the corresponding authors.

## Ethics statement

The studies involving human participants were reviewed and approved by Ethics Committee of Sapienza University of Rome (approval number 5834, 0521/2020). The patients/participants provided their written informed consent to participate in this study.

## Author contributions

CF, SR, and HS performed the experiments and analyzed the results; IQ, CM, PP, and IM provided samples from patients and clinical data; ML performed statistical analysis; HS contributed to design research and write the manuscript; CF, GS, SS, LF, AG, and AS critically reviewed the manuscript. All authors have contributed and approved the final version of the paper.

## Funding

This work was supported by grant from Ministero dell’Istruzione, dell’Università e della Ricerca (COVID-2020-12371735-Neuromed; RM12117A5D5C4A0E Ateneo 2021).

## Acknowledgments

A special thanks to all patients and healthy donors who contributed to this study.

## Conflict of interest

The authors declare that the research was conducted in the absence of any commercial or financial relationships that could be construed as a potential conflict of interest.

## Publisher’s note

All claims expressed in this article are solely those of the authors and do not necessarily represent those of their affiliated organizations, or those of the publisher, the editors and the reviewers. Any product that may be evaluated in this article, or claim that may be made by its manufacturer, is not guaranteed or endorsed by the publisher.
