# Supplementary figures and images for: Age-dependent NK cell dysfunctions in severe COVID-19 patients
Source: Front Immunol. 2022 Nov 17;13:1039120. doi: 10.3389/fimmu.2022.1039120 (PMC9713640; doi:10.3389/fimmu.2022.1039120)

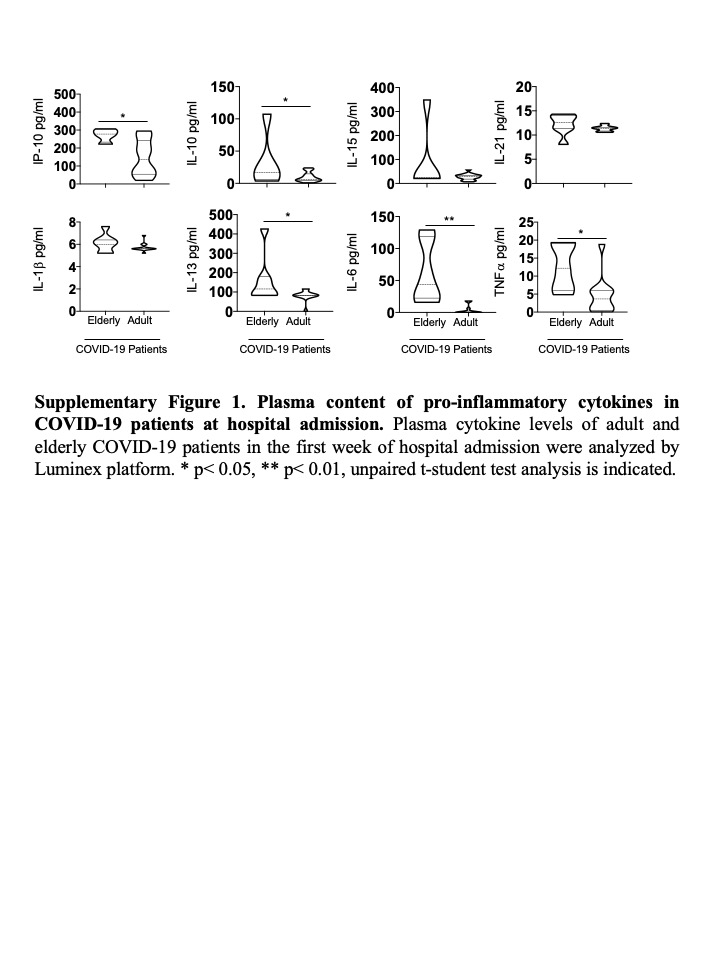

Supplement: Supplementary file 1 [file Image_1.jpeg]
