# Supplementary material for: Age-dependent NK cell dysfunctions in severe COVID-19 patients
Source: Front Immunol. 2022 Nov 17;13:1039120. doi: 10.3389/fimmu.2022.1039120 (PMC9713640; doi:10.3389/fimmu.2022.1039120)
Supplement: Supplementary file 2 [file Table_1.docx]

**Supplementary Table S1**

Patient’s clinical and demographic characteristics**.**

|  |  |  |  | **Presenting symptoms** | | | |  |
| --- | --- | --- | --- | --- | --- | --- | --- | --- |
| **ID** | **Age (y)** | **Sex** | **Co-morbidity** | **Fever** | **Cough** | **Dyspnoea** | **Gastrointestinal** |  |
| E1 | 83 | M | Hypertension/Obesity | 0 | 0 | 0 | 0 | **ELDERLY** |
| E2 | 84 | M | Hypertension | 1 | 0 | 1 | 0 |  |
| E3 | 73 | M |  | 1 | 0 | 0 | 0 |  |
| E4 | 66 | M | Hypertension//Diabetes/ | 1 | 0 | 0 | 0 |  |
| E5 | 65 | M |  | 1 | 1 | 1 | 0 |  |
| E6 | 83 | F |  | 0 | 0 | 1 | 0 |  |
| E7 | 67 | F | Obesity/COPD | 0 | 1 | 1 | 0 |  |
| E8 | 80 | M | Hypertension | 1 | 0 | 1 | 0 |  |
|  | | | | | | | |  |
|  |  |  |  |  |  |  |  |  |
| A1 | 25 | M |  | 1 | 1 | 0 | 0 | **ADULT** |
| A2 | 55 | M |  | 1 | 0 | 1 | 0 |  |
| A3 | 45 | M |  | 1 | 0 | 1 | 0 |  |
| A4 | 45 | M | Obesity | 1 | 1 | 1 | 0 |  |
| A5 | 45 | M |  | 1 | 0 | 1 | 0 |  |
| A6 | 57 | M |  | 1 | 1 | 1 | 0 |  |
| A7 | 33 | M | Obesity | 1 | 1 | 0 | 0 |  |
| A8 | 46 | M |  | 1 | 1 | 0 | 1 |  |
| A9 | 55 | M |  | 1 | 1 | 1 | 0 |  |
| A10 | 40 | M |  | 1 | 1 | 1 | 0 |  |
| A11 | 37 | M |  | 1 | 1 | 1 | 0 |  |
| A12 | 55 | M |  | 1 | 1 | 1 | 0 |  |
| A13 | 35 | M |  | 1 | 0 | 1 | 0 |  |
| A14 | 56 | F |  | 1 | 0 | 0 | 0 |  |
| A15 | 42 | M |  | 1 | 0 | 1 | 1 |  |
| A16 | 57 | M |  | 0 | 1 | 1 | 0 |  |
| A17 | 50 | M |  | 1 | 1 | 1 | 1 |  |
| A18 | 51 | F | Obesity | 1 | 0 | 0 | 0 |  |
| A19 | 45 | F |  | 1 | 0 | 0 | 0 |  |

|  | **Admission Observation** | | | | | | | | | | | |  | |
| --- | --- | --- | --- | --- | --- | --- | --- | --- | --- | --- | --- | --- | --- | --- |
| ID | **FiO2** | **pO2** | **pCO2 mmHg** | **pH** | **HCO3 mmol/L** | **P/F** | **sO2** | **Lac** | **PaO2 STANDARD** | **P/F STANDARD** | **Days from symptoms start** |  | |  |
| E1 | - | 59 | 23 | 7.30 |  | 281 | - | - | 87 |  | 10 | **ELDERLY** | |  |
| E2 | 21 | 52 | 25 | 7.58 | 11.3 |  | 98.8 | 1 | 70 | 146 | 3 |  |  |  |
| E3 | 21 | - | - |  |  |  | 99.8 | 0.9 | 113 | 129 | 11 |  |  |  |
| E4 | - | 62 | 36 | 7,47 |  |  | - | 8.9 | 53 |  | 7 |  |  |  |
| E5 | 21 | 68 | 31 | 7,49 |  | 300 | 99 | 1.9 | 98 |  | 11 |  |  |  |
| E6 | 21 | - | - |  |  | 324 | 99.4 | - | 100 |  | 1 |  |  |  |
| E7 | - | 51 | 35 | 7,35 | 22.3 |  | 100 | 0.8 | 208 | 264 | 10 |  |  |  |
| E8 | 21 | - | - |  | 23.6 | 243 | 99 | 1.2 | 80 | 253 | 3 |  |  |  |
|  | | | | | | | | | | | | |  | |
|  |  |  |  |  |  |  |  |  |  |  |  |  |  | |
| A1 | 21 | 68 | 38 | 7.46 | 27 | 324 | 99.8 | 1.5 | 105 | 308 | 12 | **ADULT** | |  |
| A2 | 21 | 59 | 31 | 7.56 | 27.8 | 281 | 98.3 | 1.3 | 73 | 210 | 6 |  |  |  |
| A3 | 21 | 64 | 25 | 7.57 | 22.9 | 305 | 100 | 2.1 | 131 | 186 | 10 |  |  |  |
| A4 | 28 | 66 | 36 |  | 25.6 | 314 | 99.5 | 0.7 | 97 | 212 | 7 |  |  |  |
| A5 | 21 | 68 | 32 | 7.52 | - | 324 | 100 | 1.3 | 97 | 260 | 5 |  |  |  |
| A6 | 21 | 64 | 33 | 7.49 | 25.1 | 305 | 99.2 | 1.1 | 89 | 250 | 0 |  |  |  |
| A7 | 21 | 66 | 34 | 7.47 | 24.7 | 314 | 100 | 1.2 | 186 | 267 | 7 |  |  |  |
| A8 | 21 | 57 | 35 | 7.48 | - |  | 99.5 | 1.2 | 113 | 232 | 9 |  |  |  |
| A9 | 21 | 60 | 29 | 7.54 | 24.8 | 286 | 98.3 | 0.9 | 70 | 198 | 10 |  |  |  |
| A10 | 21 | 64 | 31 | 7.55 | 28.8 | 305 | 100 | 2.4 | 96 | 234 | 7 |  |  |  |
| A11 | 21 | 71 | 33 | 7.50 | 27.1 | 338 | - | 1.5 | 92 | 283 | 16 |  |  |  |
| A12 | 21 | 70 | 28 | 7.52 | 25.7 | 333 | 100 | - | 122 | 238 | 10 |  |  |  |
| A13 | 21 | 60 | 33 | 7.49 | 25.1 | 285 | 97.7 | 0.9 | 115 | 230 | 9 |  |  |  |
| A14 | 21 | 54 | 37 | 7.46 | 26.6 | 257 | 100 | 3.1 | 112 | 233 | 7 |  |  |  |
| A15 | 21 | 56 | 31 | 7.51 | 26.6 | 266 | 100 | 0.8 | 154 | 195 | 8 |  |  |  |
| A16 | 21 | 73 | 29 | 7.50 | 22.6 | 348 | - | 1.3 | 113 | 261 | 3 |  |  |  |
| A17 | 21 | - | - | - | - | 314 | 100 | 1.2 | 114 | - | 5 |  |  |  |
| A18 | 21 | 66 | 34 | 7.51 | 27.1 | 343 | 99.6 | 2 | 113 | 267 | 10 |  |  |  |
| A19 | 21 | 72 | 35 | 7.46 | 24.9 | 297 | 99 | 1.2 | 96 | 303 | 5 |  |  |  |

|  | **Admission Laboratory Parameters** | | | | | | | | | |  |
| --- | --- | --- | --- | --- | --- | --- | --- | --- | --- | --- | --- |
| ID | **White Cell Count**  **(ml)** | **Neutrophils**  **(ml)** | **Lymphocytes**  **(ml)** | **Monocytes**  **(ml)** | **Eosinophilis**  **(ml)** | **Basophilis**  **(ml)** | **Platelets**  **(ml)** | **Ferritin**  **(ng/ml)** | **D-dimer**  **(ng/ml)** | **PCR**  **(mg/dl)** |  |
| E1 | 11270 | 9640 | 980 | 380 | 110 | 120 | 182 | 605 | 2570 | 20,94 | **ELDERLY** |
| E2 | 6760 | 5980 | 500 | 160 | 30 | 20 | 228 | - | 846 | 9,04 |  |
| E3 | 7090 | 5530 | 1150 | 290 | 10 | 20 | 202 | - | 1400 | 10,78 |  |
| E4 | 4110 | 3180 | 770 | 100 | 20 | 20 | 254 | 190 | 455 | 15,16 |  |
| E5 | 12700 | 11850 | 360 | 360 | 60 | 10 | 235 | 1687 | 549 | 20,37 |  |
| E6 | 15180 | 13660 | 990 | 350 | 50 | 60 | 192 | 200 | 647 | 11,49 |  |
| E7 | 3210 | 2410 | 480 | 260 | 10 | 10 | 170 | 1019 | 524 | 9,16 |  |
| E8 | 4220 | 3390 | 560 | 210 | 0 | 20 | 148 | 430 | - | 3,07 |  |
|  |  |  |  |  |  |  |  |  |  |  |  |
|  |  |  |  |  |  |  |  |  |  |  |  |
| A1 | 6280 | 5680 | 330 | 190 | 20 | 40 | 163 | 898 | 3748 | 12.95 | **ADULT** |
| A2 | 4350 | 3150 | 870 | 260 | 10 | 20 | 181 | - | 475 | 1.57 |  |
| A3 | 8340 | 6760 | 1140 | 310 | 30 | 30 | 196 | 156 | 908 | 2.04 |  |
| A4 | 9430 | 6490 | 2290 | 520 | 10 | 50 | 181 | 646 | 246 | 1.38 |  |
| A5 | 3370 | 2100 | 950 | 200 | 40 | 10 | 170 | - | 391 | 1.39 |  |
| A6 | 6220 | 5620 | 200 | 320 | 30 | 10 | 145 | 195 | 635 | 4.2 |  |
| A7 | 10160 | 8960 | 610 | 370 | 40 | 60 | 306 | - | 499 | 14.14 |  |
| A8 | 7020 | 5540 | 910 | 340 | 60 | 20 | 229 | 2139 | 984 | 7.46 |  |
| A9 | 3820 | 2480 | 1050 | 190 | 20 | 10 | 97 | 1669 | 384 | 3.01 |  |
| A10 | 8050 | 7110 | 510 | 340 | 10 | 20 | 169 | 730 | 565 | 4 |  |
| A11 | 10850 | 9230 | 920 | 550 | 0 | 3 | 245 | 1094 | 326 | 4.19 |  |
| A12 | 10860 | 9780 | 610 | 300 | 20 | 50 | 262 | 1134 | - | 12.43 |  |
| A13 | 4080 | 2950 | 800 | 230 | 10 | 20 | 141 | 1430 | - | 1.95 |  |
| A14 | 8520 | 6380 | 1480 | 560 | 30 | 30 | 233 | 394 | 4318 | 2.87 |  |
| A15 | 6640 | 5430 | 760 | 330 | 80 | 0 | 183 | - | - | 1.17 |  |
| A16 | 9850 | 9010 | 290 | 450 | 20 | 20 | 332 | 661 | 1264 | 11.46 |  |
| A17 | 6330 | 4750 | 870 | 590 | 10 | 40 | 172 | 1151 | 1063 | 5.23 |  |
| A18 | 8750 | 7750 | 460 | 420 | 20 | 10 | 298 | 637 | - | 7.5 |  |
| A19 | 4570 | 2980 | 1280 | 200 | 40 | 30 | 164 | - | 734 | 0.86 |  |

|  | **Pharmacologic Treatment** | | | | | **Outcome Data** | | |  |
| --- | --- | --- | --- | --- | --- | --- | --- | --- | --- |
| ID | **REMDESIVIR** | **SOLDESAM** | **CLEXANE** | **CLEXANE** | **ZITROMAX** | **Hospital Stay (Days)** | **Intensive Care Stay** | **Mortality** |  |
| E1 | 0 | 1 | 1 | 1 | 0 | 36 | 0 | 0 | **ELDERLY** |
| E2 | 1 | 1 | 1 | 1 | 1 | 21 | 1 | 1 |  |
| E3 | 0 | 1 | 1 | 0 | 1 | 13 | 0 | 0 |  |
| E4 | 1 | 1 | 1 | 1 | 1 | 13 | 1 | 1 |  |
| E5 | 1 | 1 | 1 | 0 | 1 | 12 | 0 | 0 |  |
| E6 | 1 | 1 | 1 | 1 | 0 | 22 | 1 | 1 |  |
| E7 | 1 | 1 | 1 | 1 | 0 | 22 | 0 | 0 |  |
| E8 | 1 | 1 | 1 | 1 | 0 | 31 | 1 | 1 |  |
|  |  |  |  |  |  |  |  |  |  |
|  |  |  |  |  |  |  |  |  |  |
| A1 | 1 | 1 | 1 | 0 | 1 | 12 | 0 | 0 | **ADULT** |
| A2 | 1 | 1 | 1 | 0 | 1 | 30 | 0 | 0 |  |
| A3 | 1 | 1 | 1 | 0 | 0 | 15 | 0 | 0 |  |
| A4 | 1 | 1 | 1 | 0 | 1 | 21 | 0 | 0 |  |
| A5 | 1 | 1 | 1 | 0 | 0 | 18 | 1 | 0 |  |
| A6 | 1 | 1 | 1 | 0 | 0 | 7 | 0 | 0 |  |
| A7 | 1 | 1 | 1 | 0 | 1 | 14 | 0 | 0 |  |
| A8 | 1 | 1 | 1 | 0 | 0 | 12 | 0 | 0 |  |
| A9 | 0 | 1 | 1 | 0 | 1 | 11 | 0 | 0 |  |
| A10 | 1 | 1 | 1 | 0 | 0 | 15 | 0 | 0 |  |
| A11 | 1 | 1 | 1 | 0 | 1 | 14 | 0 | 0 |  |
| A12 | 1 | 1 | 1 | 0 | 1 | 8 | 0 | 0 |  |
| A13 | 1 | 1 | 1 | 0 | 1 | 14 | 0 | 0 |  |
| A14 | 1 | 1 | 1 | 0 | 1 | 36 | 0 | 0 |  |
| A15 | 1 | 1 | 1 | 0 | 1 | 22 | 0 | 0 |  |
| A16 | 1 | 1 | 1 | 0 | Clarithromycin | 9 | 0 | 0 |  |
| A17 | 1 | 1 | 1 | 0 | 1 | 18 | 0 | 0 |  |
| A18 | 1 | 1 | 1 | 0 | 1 | 17 | 0 | 0 |  |
| A19 | 1 | 1 | 1 | 0 | 0 | 9 | 0 | 0 |  |

**Supplementary Table S2**

List of antibodies used for cytofluorimetric analysis.

| REAGENT | SOURCE |
| --- | --- |
|  |  |
| Antibodies |  |
| Anti-human CD45 antibody, clone HI30, BUV805 | BD Biosciences |
| Anti-human CD3 antibody, clone SK7, BV605 | BD Biosciences |
| Anti-human CD4 antibody, clone RPA-T4, BV605 | BD Biosciences |
| Anti-human CD5 antibody, clone UCHT2, BV605 | BD Biosciences |
| Anti-human CD19 antibody, clone SJ25C1, BV605 | BD Biosciences |
| Anti-human CD14 antibody, clone M5E2, BV605 | BD Biosciences |
| Anti-human CD16 antibody, clone 3G8, BV480 | BD Biosciences |
| Anti-human CD16 antibody, clone 3G8, BUV737 | BD Biosciences |
| Anti-human CD94 antibody, clone HP-3D9, BV786 | BD Biosciences |
| Anti-human CD56 antibody, clone NCAM 16.2, APC-R700 | BD Biosciences |
| Anti-human CD7 antibody, clone M-T701, PE-Cy™5 | BD Biosciences |
| Anti-human CD294 antibody, clone BM16, BUV395 | BD Biosciences |
| Anti-human CD117 antibody, clone YB5.B8, PE | BD Biosciences |
| Anti-human CD69 antibody, clone FN50, BV421 | BD Biosciences |
| Anti-human NKG2A antibody, clone 131411, BUV737 | BD Biosciences |
| Anti-human FasL antibody, clone NOK-1, PE | BD Biosciences |
| Anti-human CD107a antibody, clone H4A3, APC | BD Biosciences |
| Anti-human NKp44 antibody, clone P44-8, BUV395 | BD Biosciences |
| Anti-human SlamF7 antibody, clone 235614, APC | BD Biosciences |
| Anti-human Tim-3 antibody, clone 7D3, BB515 | BD Biosciences |
| Anti-human Tigit antibody, clone MBSA43, PE-eFluor 610 | Thermo Fisher Scientific |
| Anti-human CD200R antibody , clone OX108, PerCP-eFluor710 | Thermo Fisher Scientific |
| Anti-human CD96 antibody, clone NK92.39, PE-Cy ^TM^7 | BioLegend |
| Anti-human CD127 antibody, clone A019D5, Alexa 488 | BioLegend |
| Anti-human CD127 antibody, clone A019D5, APC-Cy7 | BioLegend |
| Anti-human CD57 antibody, clone NK-1, PE-CF594 | BD Biosciences |
| Anti-human Eomes antibody, clone WD1928, FITC | Thermo Fisher Scientific |
| Anti-human Eomes antibody, clone WD1928, PE | Thermo Fisher Scientific |
| Anti-human T-bet antibody, clone EBio4B10(4B10), PE-Cy ^TM^7 | Thermo Fisher Scientific |
| Anti-human Granzyme (Gzm) B antibody, clone GB11, FITC | BioLegend |
| Anti-human T-bet antibody, clone EBio4B10(4B10), PerCP-Cy5.5 | Thermo Fisher Scientific |
| Anti-human GATA3 antibody, clone TWAJ, eFluor 660 | Thermo Fisher Scientific |
| Anti-human Perforin (Prf) antibody, clone Dg9, PE-Cy ^TM^7 | BioLegend |
| Anti-human TNF-α antibody, clone MAb11, APC | BD Biosciences |
| Anti-human IFN-γ antibody, clone 4S.B3, PE-Cy ^TM^7 | BD Biosciences |
| Anti-human IFN-γ antibody, clone 4S.B3, PE | Thermo Fisher Scientific |
| Anti-human IFN-γ antibody, clone B27, APC | BD Biosciences |
| Anti-human CD56 antibody, clone NCAM 16.2, BV421 | BD Biosciences |

**Supplementary Table S3**

Parameters used for K-means clustering.

|  | **PATIENTS** | | | | | | | | | | | | | | | | | |
| --- | --- | --- | --- | --- | --- | --- | --- | --- | --- | --- | --- | --- | --- | --- | --- | --- | --- | --- |
| **% IFN-γ** | **A1** | **A3** | **A5** | **A6** | **A7** | **A8** | **A10** | **A11** | **A12** | **A16** | **A18** | **E1** | **E4** | **E5** | **E6** | **E3** | **E8** | **E2** |
| **CD56^high^CD16+** | 8.45 | 28.1 | 21.4 | 33.3 | 13.5 | 12.9 | 9.4 | 5.79 | 4.55 | 0 | 35.5 | 32.2 | 50 | 8.6 | 8.33 | 21.9 | 38.6 | 20 |
| **CD56^high^CD16-** | 6.26 | 44 | 11.6 | 18.1 | 10.8 | 11 | 8.9 | 11.6 | 3.12 | 0 | 49.5 | 20.9 | 57.6 | 9.76 | 0 | 28.8 | 29.1 | 71.4 |
| **CD56^low^CD16^+^** | 13.2 | 11.2 | 10.8 | 16.7 | 10.9 | 1.76 | 6.51 | 2.73 | 2.38 | 9.27 | 31.1 | 27.9 | 35.7 | 5.09 | 3.45 | 14.1 | 14 | 10 |
| **CD56^low^CD16^low^** | 29.7 | 50.7 | 25.5 | 29.4 | 18.3 | 8.69 | 8.93 | 10,2 | 11.5 | 43.5 | 30.7 | 47.6 | 44.4 | 17.3 | 18.8 | 34.2 | 26.6 | 35.5 |
| **CD56^-^** | 13.2 | 14.7 |  | 23.8 | 16.1 | 6.57 | 8 | 3.69 | 5.81 | 6.13 | 19.2 | 25.1 | 15.8 | 5.94 | 2.09 | 14.9 | 9.98 | 15.9 |
|  |  |  |  |  |  |  |  |  |  |  |  |  |  |  |  |  |  |  |
| **Day of hospitalization** | 12 | 15 | 18 | 7 | 14 | 12 | 15 | 14 | 8 | 9 | 17 | 36 | 13 | 12 | 22 | 13 | 31 | 21 |
